# The Assessment of Knowledge, Awareness and Practice Regarding Diverticulitis and Its Risk Factors Among the Population of Saudi Arabia

**DOI:** 10.7759/cureus.60124

**Published:** 2024-05-11

**Authors:** Medhat Taha, Anas T Fakieh, Abdulrahman M Alhazmi, Albaraa J Khiami, Emad A Alasmari, Salman S Alharbi, Muteb H Almajnoni

**Affiliations:** 1 Department of Anatomy, Umm Al-Qura University, Al-Qunfudhah, SAU; 2 College of Medicine, Umm Al-Qura University, Al-Qunfudhah, SAU

**Keywords:** saudi arabia, risk factors, awareness, knowledge, incidence, diverticular disease, diverticulitis

## Abstract

Background

The term "diverticula" refers to the existence of diverticula in the gastrointestinal tract but is mainly located in the sigmoid colon and is used to describe colonic diverticulosis. Diverticula, which are sac-like protrusions in the wall of the large bowel, are becoming more prevalent globally, in both developed and developing nations. This increase in occurrence is primarily attributed to changes in dietary and lifestyle patterns. Raising public awareness can potentially contribute to a decrease in the incidence of the disease and its associated complications.

Aim

This study aims to assess knowledge and awareness levels among the Saudi Arabian population regarding diverticulitis and its risk factors.

Methods

A descriptive cross-sectional study was conducted in Saudi Arabia between 1st January 2024 to 1st April 2024 using an online questionnaire for data collection. The target population consists of individuals who are between 18 years and 45, in Saudi Arabia without a history of diverticulitis. The study questionnaire covered participants' demographic (Western, Central, Southern, Eastern, Northern) regions, knowledge, awareness and practice of diverticulitis.

Results

A total of 548 eligible participants completed the study questionnaire, most of them (80.3%; 395) were from the Western region including Mecca, Medina and Jeddah. Participants' ages ranged from 18 to more than 40 years with a mean age of 30.5 ± 11.9 years old. A greater percentage (72.3%) of the participants were males compared to the percentage of females, which was 27.7%. The vast majority of the study participants had an inadequate knowledge level about diverticulitis (85.9%; 471) while only 31 (5.7%) had adequate knowledge and awareness about the disease. The most reported sources of information included study courses (6.4%), media (5.3%), and physicians (4.7%) while most respondents (83.6%) had no source.

Conclusion

In conclusion, aside from preventive strategies, the current study found that the public knew very little about diverticulitis, including its risk factors, clinical presentation, and diagnostic process. The two significant predictors of public awareness level were age and doctors as information sources.

## Introduction

Diverticula, characterized as pouch-like bulges in the wall of the gastrointestinal tract, the most frequently encountered anatomical modification, is in the colon, these sac-like protrusions are highly prevalent, making them the predominant structural change observed in the colon [1]. The word diverticula describes colonic diverticulosis and denotes the presence of diverticula in the gastrointestinal tract [2]. Changes in diet and lifestyle are playing a significant role in the growing occurrence of diverticulosis globally, affecting both developed and developing countries [3,4]. The exact underlying mechanisms of diverticular disease are still not fully comprehended. Several factors have been proposed to contribute to its development, including the structure of the colonic wall, the motility of the colon, age, dietary patterns and fiber consumption, obesity and physical activity, as well as genetic susceptibility [5,6].

Older adults frequently have diverticulosis, which affects 50% of those over 60 [7]. In developed nations, diverticulosis affects up to two-thirds of people over the age of 18, and the incidence is still rising [8,9]. Although symptoms are experienced by 20% of patients with colonic diverticulosis, most individuals with the illness may be asymptomatic [10]. According to age-related increases in prevalence, diverticulosis affects 70% of those who are at least 80 years old. Every year, diverticular disease is blamed for more than 300,000 hospital admissions, 1.5 million inpatient treatment days, and $2.4 billion in direct expenses [11]. Based on a retrospective study conducted in Saudi Arabia with a mean age of 60 years, it was observed that the prevalence of colonic diverticulosis in the population was approximately 7.4%. Remarkably, this finding gains particular significance when considering that approximately half of individuals aged 60 years old are affected by this condition [12].

Although recent research indicates that diverticulosis and diverticulitis are becoming increasingly more common, especially in younger individuals; they are extremely rare conditions before the age of 30 and become more common with higher age [13]. Moreover, most previous studies that relied on registry-level data included hospitalized patients, who frequently represent more severe cases. A recent study found that eating more fiber as part of a healthy lifestyle significantly lowers the incidence of diverticulitis, which can range from mild cases that can be managed at home to more serious cases that need to be hospitalized [14]. There are few research studies assessing the knowledge of diverticulitis reasons and problems globally. Therefore, the current study aims to close this research gap. The primary aim of our study is to assess the knowledge and awareness of diverticulitis among the population of Saudi Arabia.

## Materials and methods

Study design and participants

A descriptive cross-sectional study was applied to evaluate the levels of knowledge and awareness regarding diverticulitis within the Saudi population. An online anonymous questionnaire was precisely developed and then distributed among a representative sample of the populace from 1st January 2024 to 1st April 2024. The study included Saudi Arabian residents of both genders aged 18 and above. However, individuals who did not meet the specified criteria, specifically Saudi Arabian residents who were below 18 years old, and participants diagnosed with diverticulitis, who had incomplete survey records, were not included in the study. The sample was calculated using the Raosoft calculator (Raosoft, Inc., Seattle, WA) was used to determine the sample size. According to the most recent report from 2022, 34,110,821 people called Saudi Arabia home. Three hundred and eighty-four is the bare minimum sample size that is anticipated when a 95%confidence level (95% CI) and 5% margin of error are used.

Data collection

A pre-structured online questionnaire was utilized for data collection. The self-reported online questionnaire was distributed in Arabic and was shared via various social media platforms (Twitter and WhatsApp) to friends, family members and colleagues to assist with the distribution of the survey. The questionnaire included many sections. The first section contains consent, the second includes sociodemographic questions, the third contains questions to assess the knowledge regarding diverticulitis, and the last contains questions to assess the awareness regarding diverticulitis. Most of the questions will be on Three-Point Scales (Yes, No, I don’t know). This questionnaire takes an average of four to five minutes to complete. The study questionnaire was made available until no further responses were received or until the desired sample size was reached.

Methods

Participants’ knowledge regarding diverticulitis was assessed based on their responses to 12 items. Each correct answer was assigned one and incorrect responses were assigned zero. An overall knowledge score (range 0 to 16) was computed by summing the correct answers, and a percent score (range 0 to 100) was calculated to facilitate the interpretation of scores. Knowledge levels were categorized as inadequate knowledge (0 to 50), moderate knowledge (51 to 75) and adequate knowledge (>75).

Data analysis

**Figure 1:**
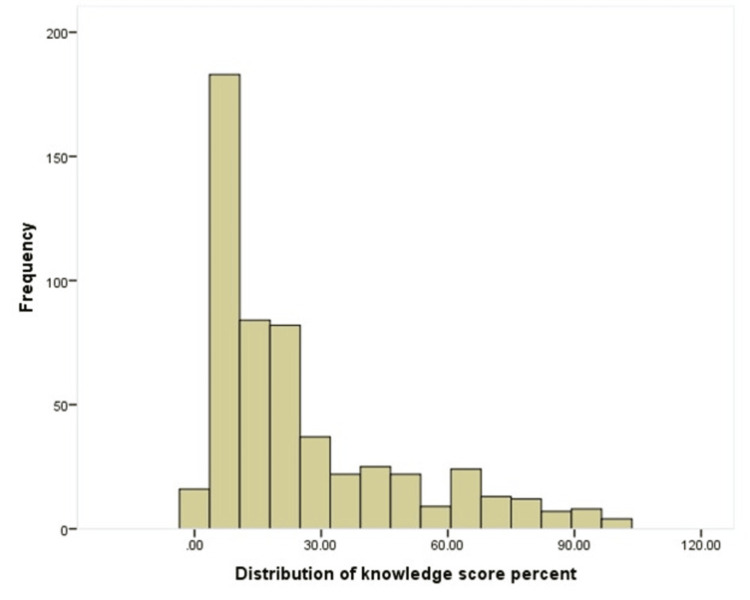
A histogram depicting the frequency distribution of the knowledge score.

The data were collected, reviewed and then fed to Statistical Package for the Social Sciences (IBM SPSS Statistics for Windows, IBM Corp., Version 26.0, Armonk, NY). All statistical methods used were two-tailed with an alpha level of 0.05 considering significance if the p-value is less than or equal to 0.05. An overall knowledge score was computed by summing the correct answers, and a percent score (range 0 to 100) was calculated to facilitate the interpretation of scores. Knowledge levels were categorized as inadequate knowledge (0% to 50%), moderate knowledge (51% to 75%) and adequate knowledge (>75%). The shape of the distribution of the knowledge score was tested using a Shapiro-Wilk test, which showed a statistical significance (p < 0.001), demonstrating a skewed distribution. This was confirmed by a visual representation of the relevant histogram (Figure 1). Consequently, non-parametric testing was used for data analysis. Descriptive analysis for categorical data was done using frequencies and percentages, whereas numerical data were presented as median and interquartile range (IQR). Also, participants' knowledge and awareness, and practice about diverticulitis were tabulated while the overall knowledge level was graphed. Cross tabulation for showing factors associated with participants' knowledge about diverticulitis using Kruskal-Wallis and Man-Whitney tests for comparing median score percent. The multivariable generalized linear regression analysis to assess the independent predictors of knowledge regarding diverticulitis using the significantly associated variables as independent variables, and the percent knowledge score as a dependent variable. Results were presented as beta coefficients and 95% CIs. Statistical significance was set at p<0.05.

## Results

A total of 548 eligible participants completed the study questionnaire, most of them (80.3%; 395) were from the Western region. Participants' ages ranged from 18 to more than 40 years with a mean age of 30.5 ± 11.9 years old. A greater percentage (72.3%) of the participants were males compared to the percentage of females, which was (27.7%). A total of 538 (98.2%) were Saudi and 488 (89.1%) were residents of the city. Considering educational level, 351 (64.1%) were university graduates, 108 (19.7%) had a secondary level of education and 25 (4.6%) had a post-graduate degree. About 313 (57.1%) were 150-169 cm in height and 182 (33.2%) were 170-180 cm. As for weight in Kg, 220 (40.1%) weighted 50-69 Kg and 261 (47.6%) weighted 70-110 Kg (Table 1).

**Table 1:** Personal characteristics of study participants, Saudi Arabia (n=548)

Personal Characteristics		No	%
Region	Central Region	44	8.9%
	Northern Region	12	2.4%
	Eastern Region	19	3.9%
	Western Region	395	80.3%
	Southern Region	22	4.5%
Gender	Male	396	72.3%
	Female	152	27.7%
Age in years	<20	32	5.8%
	20-30	269	49.1%
	30-40	107	19.5%
	>40	140	25.5%
Height in cm	<150	24	4.4%
	150-169	313	57.1%
	170-180	182	33.2%
	>180	29	5.3%
Weight in Kg	<50	40	7.3%
	50-69	220	40.1%
	70-110	261	47.6%
	>110	27	4.9%
Educational level	Below secondary	16	2.9%
	Secondary	108	19.7%
	Diploma	48	8.8%
	University	351	64.1%
	Post-graduate	25	4.6%
Nationality	Saudi	538	98.2%
	Non-Saudi	10	1.8%
Residence	City	488	89.1%
	Village	60	10.9%

Table 2 shows that only 16.4% of the respondents know about diverticulitis, 23.7% know that there are other names for diverticulitis, and 16.1% think diverticulitis is a serious disease. Precisely 48.2% of individuals believe that there are different classifications or categories of diverticulitis, 44% think that a family history of diverticulitis is an important risk factor, and 36.5% incorrectly reported that diverticulitis affects people under 40 years old. Regarding risk factors, the most reported were low in fiber and rich in red meat diet (13%), smoking (8.4%), obesity (5.3%), and lack of exercise (3.5%) but the vast majority of the participants don’t know about risk factors. Considering symptoms, abdominal pain was the most known (24.1%), followed by nausea and vomiting (16.6%), fever (13.9%), and constipation (12.4%) while 73% don’t know about clinical symptoms. A total of 15% know preventive measures or lifestyle changes that can help reduce your risk of developing diverticulitis, 19.7% know about potential complications associated with severe or untreated diverticulitis, and 7.8% know the difference between diverticulosis and diverticulitis. CT scan was the most reported diagnostic method used to diagnose diverticulitis (10.4%) while 80.8% didn’t know about diverticulitis diagnosis.

**Table 2:** Study participants' knowledge and awareness towards diverticulitis and its risk factors, Saudi Arabia

	Knowledge and Awareness	No	%
Do you know about diverticulitis?	Yes	90	16.4%
	No	458	83.6%
Are there other names for diverticulitis?	Yes	130	23.7%
	No	418	76.3%
Do you think diverticulitis is a serious disease?	Yes	88	16.1%
	No	46	8.4%
	I don't know	414	75.5%
Do you think there are types of diverticulitis?	Yes	264	48.2%
	No	284	51.8%
Do you think that a family history of diverticulitis is an important risk factor?	Yes	241	44.0%
	No	307	56.0%
Diverticulitis affects people under 40 years old?	Yes	200	36.5%
	No	348	63.5%
The most significant risk factors associated with developing diverticulitis?	Low in fiber and rich in red meat diet	71	13.0%
	Smoking	46	8.4%
	Obesity	29	5.3%
	Lack of exercise	19	3.5%
	I don't know	383	69.9%
Symptoms of diverticulitis?	Abdominal pain	132	24.1%
	Nausea and vomiting	91	16.6%
	Fever	76	13.9%
	Constipation	68	12.4%
	I don't know	400	73.0%
Know preventive measures or lifestyle changes that can help reduce your risk of developing diverticulitis?	Yes	82	15.0%
	No	64	11.7%
	I don't know	402	73.4%
Do you know the difference between diverticulosis and diverticulitis?	Yes	43	7.8%
	No	101	18.4%
	I don't know	404	73.7%
Are there any potential complications that associated with severe or untreated diverticulitis?	Yes	108	19.7%
	No	22	4.0%
	I don't know	418	76.3%
Are you aware of any diagnostic methods or tests used to diagnose diverticulitis?	CT scan	57	10.4%
	Abdominal X-Ray	23	4.2%
	Clinical diagnosis	18	3.3%
	Abdominal US	7	1.3%
	I don't know	443	80.8%

**Figure 2:**
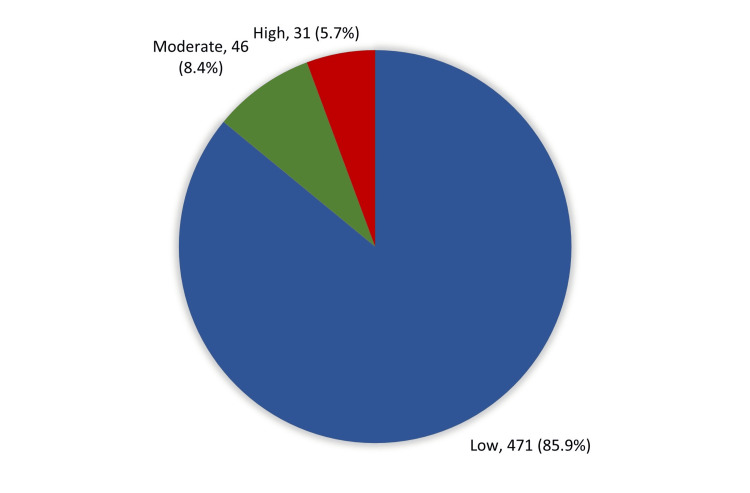
Overall knowledge and awareness level regarding diverticulitis among study participants

As for the overall knowledge and awareness level (Figure 2), the vast majority of the study participants had an inadequate knowledge level about diverticulitis (85.9%; 471) while only 31 (5.7%) had adequate knowledge and awareness about the disease.

**Figure 3:**
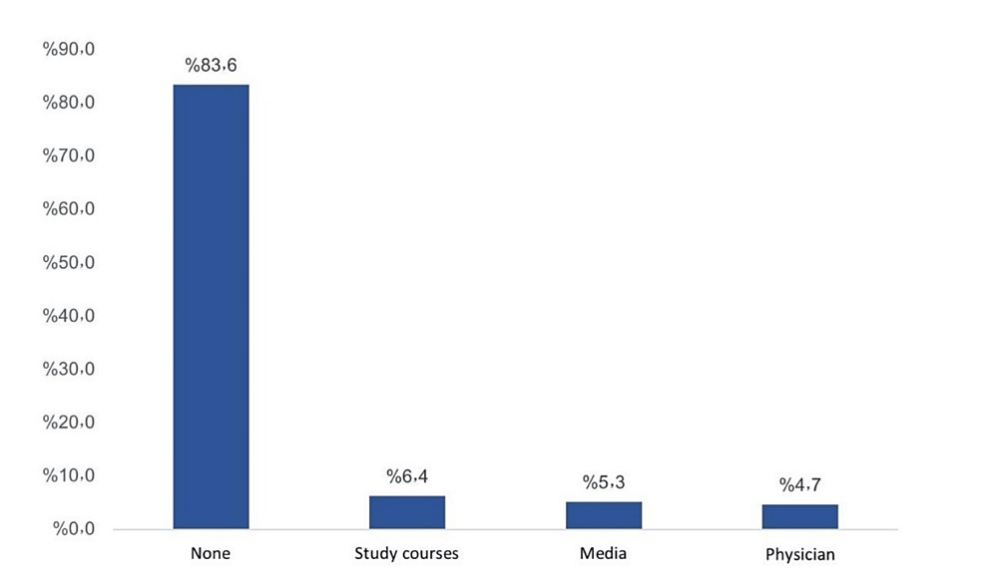
Source of information about diverticulitis among study participants

The most reported sources of information included study courses (6.4%), media (5.3%), and physicians (4.7%) while most respondents (83.6%) had no source (Figure 3).

Out of the total participants, precisely 26 individuals, accounting for 4.7% of the sample, reported their active involvement in diverticulitis awareness campaigns or events. As for the probability of seeking medical care if experienced symptoms that may be related to diverticulitis, 109 (19.9%) rated the probability 1 out of 5, 174 (31.8%) rated 5 out of 5, and 154 (28.1%) rated 5 out of 5 (Table 3).

**Table 3:** Participants practice and training regarding diverticulitis disease

Practice	No	%
How likely you are to seek medical care if you experience symptoms that may be related to diverticulitis?		
1/5	109	19.9%
2/5	30	5.5%
3/5	174	31.8%
4/5	81	14.8%
5/5	154	28.1%
Have you ever participated in any diverticulitis awareness campaigns or events?		
Yes	26	4.7%
No	522	95.3%

The differences in knowledge scores based on sociodemographic characteristics and participants’ practices for detecting diverticulitis were illustrated in the table where the median knowledge score percent was 28.6 (21.4, 57.1) among young aged participants versus 7.1 (6.9, 14.3) for old aged respondents (P<0.001). Also, the median knowledge score percent was 28.6 (14.3, 50.0) for participants less than 50 Kg compared to 14.3 (7.1, 35.7) for those weighted more than 110 Kg (P=.014). Additionally, the median score percent was 35.7 (21.4, 64.3) for those who participated in any diverticulitis awareness campaigns or events compared to 14.3 (14.0, 21.4) for others (P<0.001). The median knowledge score percent was 71.4 (69.3, 85.7) for those who had their information from physicians versus 50 (42.8, 64.3) for media (P=0.009) (Table 4).

**Table 4:** Differences in knowledge scores based on sociodemographic characteristics and participants’ practices for detecting diverticulitis P: Kruskal-Wallis test; #: Mann-Whitney test; * P <0.05 (significant)

Factors		Median (IQR)	p-value
Region	Central Region	21.4 (21.4, 42.9)	0.227
	Northern Region	35.7 (7.1, 78.6)	
	Eastern Region	14.3 (7.1, 28.6)	
	Western Region	14.3 (14.0, 21.4)	
	Southern Region	17.9 (7.171.4)	
Age in years	<20	28.6 (21.4, 57.1)	<0.001*
	20-30	21.4 (20.1, 35.7)	
	30-40	14.3 (14.0, 21.4)	
	>40	7.1 (6.9, 14.3)	
Gender	Male	14.3 (14.0, 21.4)	0.125^#^
	Female	21.4 (21.0, 28.6)	
Height in cm	<150	17.9 (14.3, 35.7)	0.264
	150-169	14.3 (14.0, 21.4)	
	170-180	21.4 (20.3, 35.7)	
	>180	14.3 (14.0, 28.6)	
Weight in Kg	<50	28.6 (14.3, 50.0)	0.014*
	50-69	17.9 (14.2, 21.4)	
	70-110	14.3 (13.8, 21.4)	
	>110	14.3 (7.1, 35.7)	
Educational level	Below secondary	17.9 (7.1, 28.6)	0.178
	Secondary	21.4 (20.2, 35.7)	
	Diploma	14.3 (7.1, 21.4)	
	University	14.3 (13.8, 21.4)	
	Post-graduate	14.3 (13.6, 28.6)	
Nationality	Saudi	14.3 (13.9, 21.4)	0.651^#^
	Non-Saudi	21.4 (7.1, 64.3)	
Residence	City	14.3 (13.9, 21.4)	0.610^#^
	Village	14.3 (14.0, 28.6)	
Have you ever participated in any diverticulitis awareness campaigns or events?	Yes	35.7 (21.4, 64.3)	<0.001*^#^
	No	14.3 (14.0, 21.4)	
Source of information	Physician	71.4 (69.3, 85.7)	0.009*
	Study courses	71.4 (69.2, 85.7)	
	Media	50 (42.8, 64.3)	

Multiple linear regression models for predictors of high knowledge scores of diverticulitis in (Table 5) revealed that among all included factors, only participants' age and physician as a source of information were significantly associated with high knowledge scores. The score showed a 2.5% increase for each additional year of age while those who gained their information from a physician had a 12% higher knowledge score level than others.

**Table 5:** Generalized linear regression analysis model for predictors of high knowledge scores of diverticulitis among study participants B: Regression co-efficient; SE: standard error; * P <0.05 (significant)

Factors	Unstandardized Coefficients		Standardized Coefficients	t	p-value
	B	SE	Beta		
Central region vs. others	1.0	2.7	0.05	-0.39	0.701
Female vs. male	4.4	6.9	0.08	0.63	0.530
Age in years	2.5	0.25	0.07	2.98	0.049*
Height in cm	-9.2	5.7	-0.23	-1.62	0.110
Weight in Kg	4.5	4.2	0.14	1.08	0.283
High education vs. low education	2.7	2.8	0.11	0.95	0.347
Saudi vs. non-Saudi	17.6	14.0	0.15	1.25	0.215
Urban vs. Rural residence	8.5	7.8	0.12	1.09	0.278
Have you ever participated in any diverticulitis awareness campaigns or events?	8.4	9.1	0.10	0.92	0.359
Physician source of information vs. others	12.0	3.5	0.42	3.46	0.001*

## Discussion

The primary aim of our study is to determine the general population’s knowledge and awareness of diverticulitis among the population in the Kingdom of Saudi Arabia. Diverticulitis arises from the inflammation of tiny pockets within the large intestinal wall, typically without a clearly identified cause [15,16]. Currently, the specific pathogenic pathways that lead to the formation of diverticula in the colon are not clearly understood. Many theories exist, pertaining to motility, food, genetics, microbiota, and inflammation, among others [17].

The current study showed that the public knowledge and awareness regarding diverticulitis was very poor in total and for its related risk factors. To provide a more comprehensive explanation, it is evident that a minority, specifically less than 20% of the participants, possess an understanding of diverticulitis and acknowledge its significance as a serious medical condition. About one-fourth of them know that there are other names for diverticulitis but about half of the respondents think there are types of diverticulitis but less percent know that a family history of diverticulitis is an important risk factor. About one-third incorrectly reported that diverticulitis affects people under 40 years old. Diverticular disease was first attributed to elderly patients, with a maximal incidence in patients above 70 years old [18]. Recent medical literature shows, however, an increase in diverticulosis incidence in young patients. The most relevant increase was encountered in the group aged 18-44, where the incidence per 1,000 pop. rose from 0.151 to 0.251 in only seven years [19]. A prospective study of 207 individuals with diverticulitis who were admitted to a single facility between the ages of 27 and 92 revealed that 25 of the patients were under the age of 45 [20].

Regarding risk factors, the most reported were low in fiber and rich in red meat diet, smoking, obesity, and lack of exercise but the vast majority of the participants don’t know about risk factors. According to an earlier study by Painter and Burkitt [21], the frequency of diverticulosis differs dramatically between Western and African populations based on differences in fiber consumption. The role of fiber intake was questioned in two recent colonoscopy-based investigations. When using the Mini Dietary Assessment index to compare patients with and without diverticulosis, Song and colleagues did not identify any variations in dietary fiber scores [22]. Surprisingly, two recent investigations carried out in the USA and Japan discovered a favorable correlation between the occurrence of diverticulosis and dietary fiber consumption [23,24]. Smoking was also reported as a risk factor for diverticulitis [25,26], obesity with lack of physical activity [27,28].

Considering symptoms, the most reported included abdominal pain, nausea and vomiting, fever, and constipation but about three-fourths don’t know about clinical symptoms. Acute diverticulitis can manifest as either persistent, severe or mildly intermittent stomach pain. Fever and altered bowel habits are common systemic signs. About 50% of patients complain of constipation, while 25% to 35% report diarrhea [29]. Urinary problems, nausea, and vomiting are other symptoms also reported in the literature [16]. Few respondents know preventive measures or lifestyle changes that can help reduce your risk of developing diverticulitis, and also awareness about potential complications associated with severe or untreated diverticulitis was very low. The current study also showed that few participants know the difference between diverticulosis and diverticulitis. Diverticulosis is the formation of abnormal pouches in the bowel wall. Diverticulitis is inflammation or infection of these abnormal pouches. Together, these conditions are known as diverticular disease [30]. CT scan was the most reported diagnostic method used to diagnose diverticulitis while the vast majority of the respondents don’t know about diverticulitis diagnosis.

The lack of previous studies evaluating public awareness of diverticulitis renders any comparison of pioneer results irrelevant to the current study. Brière et al. [31] assessed suboptimal knowledge about diverticulitis among physicians. In Saudi Arabia, AlHussaini et al. [32] documented that 20% of the medical students reported diverticulitis as one of the most common causes of lower gastrointestinal bleeding.

The most reported sources of information were study courses, media then physicians indicating insufficient rules for physicians in improving public awareness. The study provided greater clarity by highlighting the substantial impact of physicians as an information source, in addition to considering the age of participants, in influencing their knowledge about diverticulitis. Our study has a few limitations that should be acknowledged. Firstly, since our study is cross-sectional in nature, we cannot determine causal relationships between variables, which restricts our ability to investigate cause-and-effect associations. Secondly, our study specifically concentrated on the population of Saudi Arabia, with the majority of participants coming from the Western and Central regions. This geographical focus may affect the applicability of our findings to other populations. The cross-sectional studies often rely on self-reported data, which can be subject to recall bias. Participants may have difficulty accurately recalling past events or behaviors, leading to misclassification and affecting the validity of the results. However, it is important to note that the survey was conducted anonymously and on a voluntary basis, ensuring the accuracy of the captured clinical data. Also, large-scale studies are recommended to comprehensively assess the disease incidence, risk factors and associated clinical profile in Saudi Arabia.

## Conclusions

In conclusion, the current study revealed the inadequate level of public knowledge about diverticulitis in all aspects including its risk factors, clinical presentation and diagnostic procedure besides their preventive measures. Old age and physicians as sources of information were the most significant predictors of public awareness level. More effort should be paid to improve public awareness about colon diseases in general and diverticular disorders in specific with the role of lifestyle modification and dietary habits in lowering diverticulitis attacks. This can be achieved through health education campaigns, media and more effort paid by physicians.
